# Supplementary material for: Corn Cob Char as Catalyst Support for Developing Carbon Nanotubes from Waste Polypropylene Plastics: Comparison of Activation Techniques
Source: Polymers (Basel). 2022 Jul 16;14(14):2898. doi: 10.3390/polym14142898 (PMC9318988; doi:10.3390/polym14142898)

Supporting Information

on

Corn cob char as catalyst support for developing carbon nanotubes from waste polypropylene plastics: Comparison of activation techniques.

Figure S1. Energy-dispersive X-ray spectroscopy (EDXS) result of all calcined catalysts: (A) NiMo/AC_0; (B) NiMo/AC_X and (C) NiMo/AC_T.

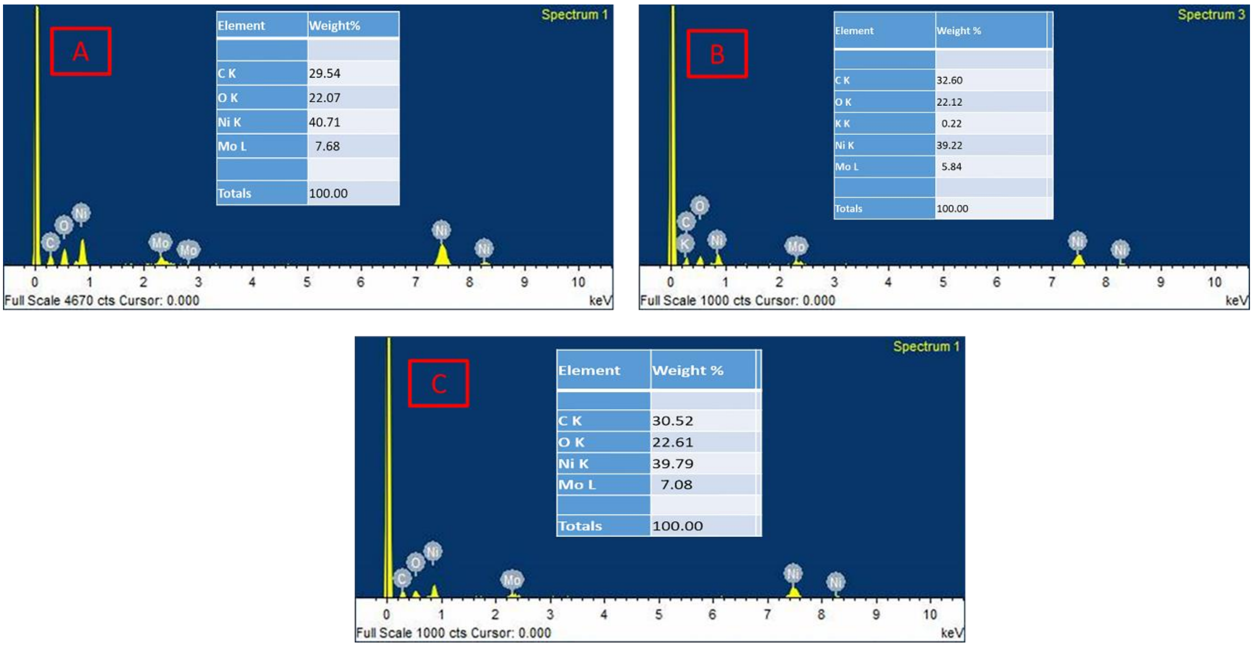

Table S1. Energy-dispersive X-ray spectroscopy (EDXS) result of all as-synthesized carbon nanotubes (CNTs) deposited on the catalysts surface.

Element	CNT_0 (weight %)	CNT_X (weight %)	CNT_T (weight %)
**C**	69.69 +/- 0.31	70.77 +/- 0.25	61.22 +/- 0.38
**K**	-	0.10 +/- 0.04	-
**Mo**	6.18 +/- 0.23	3.38 +/- 0.14	4.51 +/- 0.34
**Ni**	23.10 +/- 0.22	24.74 +/- 0.20	31.85 +/- 0.32
**O**	1.03 +/- 0.12	1.11 +/- 0.18	2.42 +/- 0.08

Figure S2. Deconvoluted Raman spectra of as-produced CNMs: CNT_X (A), CNT_0 (B) and CNT_X (C)

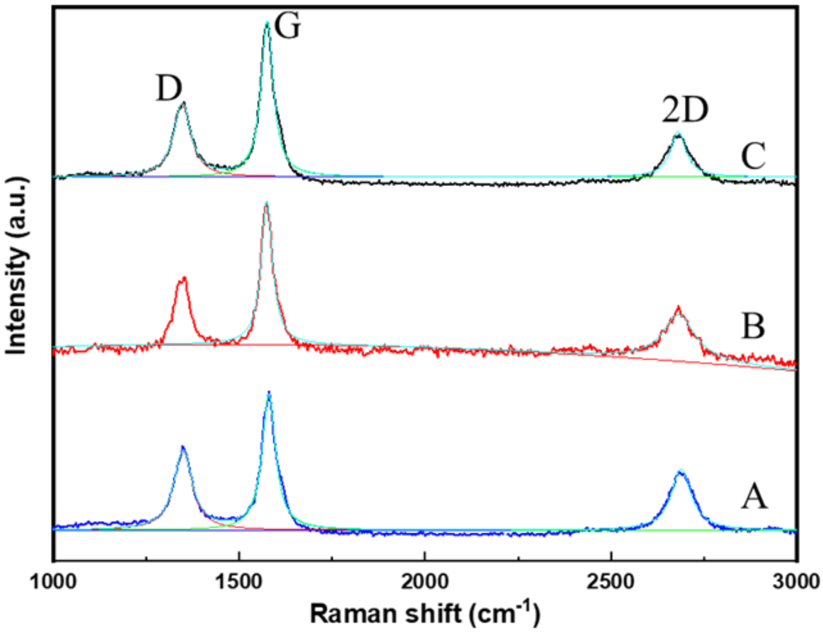

Supplement: Supplementary file 1 [file polymers-14-02898-s001.zip › polymers-1678421-supplementary.pdf]
